# CLN7/MFSD8 may be an important factor for SARS-CoV-2 cell entry

**DOI:** 10.1016/j.isci.2022.105082

**Published:** 2022-09-06

**Authors:** Elena-Sofia Heinl, Sebastian Lorenz, Barbara Schmidt, Nouf Nasser M Laqtom, Joseph R. Mazzulli, Laetitia Francelle, Timothy W. Yu, Benjamin Greenberg, Stephan Storch, Ines Tegtmeier, Helga Othmen, Katja Maurer, Malin Steinfurth, Ralph Witzgall, Vladimir Milenkovic, Christian H. Wetzel, Markus Reichold

**Affiliations:** 1 Medical Cell Biology, University Regensburg, 93053 Regensburg, Germany; 2 Institute of Clinical Microbiology and Hygiene, University of Regensburg, 93053 Regensburg, Germany; 3 Departments of Chemical Engineering and Genetics, Stanford University, Stanford, CA 94305, USA; 4 The Ken and Ruth Davee Department of Neurology, Northwestern University Feinberg School of Medicine, Chicago, IL 60611, USA; 5 Division of Genetics and Genomics, Boston Children’s Hospital, Boston, MA, USA; 6 Department of Pediatrics, Harvard Medical School, Boston, MA, USA; 7 Department of Neurology, UT Southwestern Medical Center, Dallas, TX, USA; 8 Department of Psychiatry and Psychotherapy, University of Regensburg, 93053 Regensburg, Germany; 9 Institute for Molecular and Cellular Anatomy, University Regensburg, 93053 Regensburg, Germany; 10 Children’s Hospital Biochemistry, University Medical Center Hamburg Eppendorf, 20246 Hamburg, Germany

**Keywords:** Virology, Cell biology

## Abstract

The SARS-CoV-2 virus has triggered a worldwide pandemic. According to the BioGrid database, CLN7 (MFSD8) is thought to interact with several viral proteins. The aim of this work was to investigate a possible involvement of CLN7 in the infection process. Experiments on a CLN7-deficient HEK293T cell line exhibited a 90% reduced viral load compared to wild-type cells. This observation may be linked to the finding that CLN7 ko cells have a significantly reduced GM1 content in their cell membrane. GM1 is found highly enriched in lipid rafts, which are thought to play an important role in SARS-CoV-2 infection. In contrast, overexpression of CLN7 led to an increase in viral load. This study provides evidence that CLN7 is involved in SARS-CoV-2 infection. This makes it a potential pharmacological target for drug development against COVID-19. Furthermore, it provides insights into the physiological function of CLN7 where still only little is known about.

## Highlights

• CLN7 knockout protects cells from SARS-CoV-2 infection
• CLN7 knockout leads to a strong reduction of cellular GM1 content
• A reduced GM1 content might lead to a reduced lipid raft function in CLN7-deficient cells

## Graphical abstract

**Figure:**
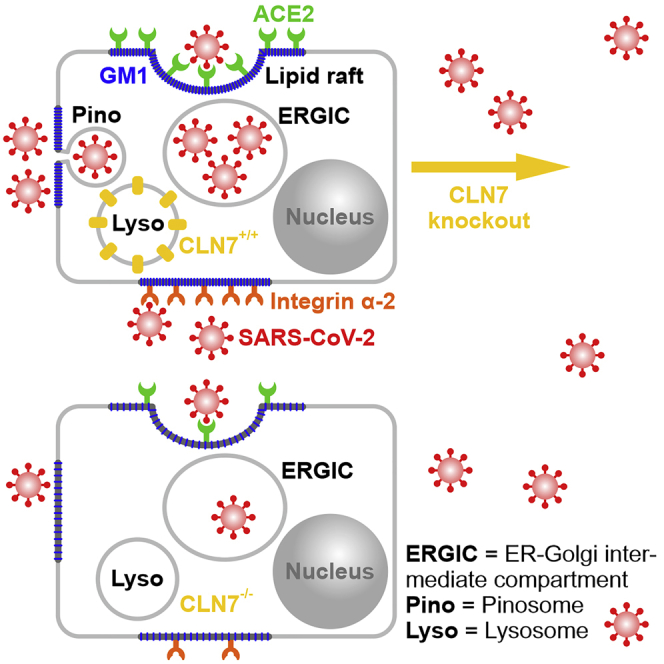

## Introduction

In 2019, the novel coronavirus SARS-CoV-2 has triggered a global pandemic. Since then, an increasing number of factors were identified which are responsible for virus uptake, replication, and exocytosis. As with all coronaviruses, the spike protein (S) plays a critical role in the initial binding of viruses to target cells as well as their internalization. The S protein consists of two functional domains, an S1 domain (receptorbinding domain, RBD) for binding to the receptor of the target cell and an S2 domain, which mediates the fusion of the virus with the cell membrane. First, the virus binds to the ACE2 receptor (angiotensin-converting enzyme 2) via the S1 domain. Then, the S1 domain is cleaved by the proteases furin and TMPRSS2, enabling the S2 domain to initiate fusion of the viral capsid with the cell membrane of the target cell. Thus, ACE2, furin, and TMPRSS2 are considered the most important proteins for viral uptake (Hoffmann et al., 2020).

In addition, other proteins have also been found to be involved in SARS-CoV-2 internalization and may serve as alternative receptors. The protein neuropilin-1, which is expressed in neurons, is thought to serve as a cofactor of SARS-CoV-2 entry into target cells, including the CNS (Cantuti-Castelvetri et al., 2020; Daly et al., 2020). This may explain the numerous central symptoms of patients with COVID-19, although neurons express little ACE2. Another potential receptor is the protein CD147 (basigin or extracellular matrix metalloproteinase inducer, EMMPRIN) (Wang et al., 2020), which is also used by the malaria pathogen as a receptor for erythrocytes. Proteins of the integrin family (Carvacho and Piesche, 2021; Park et al., 2021; Sigrist et al., 2020; Simons et al., 2021) are also thought to be responsible for SARS-CoV-2 attachment to target cells. In addition to surface proteins, other cellular structures were identified, which promote virus uptake. These include gangliosides, which are clustered in lipid rafts of the cell membrane (Fantini et al., 2020b). Ganglioside GM1 has been described to act directly as a cofactor for binding of the virus to the cell membrane (Fantini et al., 2020a). Little is yet known about the replication of the virus. The new virus particles are thought to be assembled in the “ER-Golgi intermediate compartment” (ERGIC) or Golgi apparatus (Malik, 2020). From there, large vesicles lace off, generally containing multiple viruses. These vesicles fuse with the plasma membrane, releasing the viruses from the cell (Brahim Belhaouari et al., 2020).

Via the online database BioGrid, the CLN7 protein (also called MFSD8) was recently published to interact with several SARS-CoV-2 proteins (https://thebiogrid.org/129168/summary/homo-sapiens/mfsd8.html). These include the accessory proteins ORF7A, ORF7B, ORF14, NSP4, and NSP6, as well as the structural proteins E (envelope) and S (spike). The CLN7 protein is an integral lysosomal transmembrane protein with 12 membrane-spanning domains. For a long time, it was thought to be a member of the “major facilitator superfamily” (MFS) based on sequence homologies. This group includes all secondary active transporters (permeases) that transport small molecules across the cell membrane along an established gradient under indirect energy consumption (Pao et al., 1998; Siintola et al., 2007). However, recent studies have shown that CLN7 is a lysosomal chloride channel. The physiological roles of CLN7 here are to regulate lysosomal pH, lysosomal membrane potential, and release of lysosomal Ca^2+^ via TRPML1 (Wang et al., 2021).

Mutations in the CLN7 gene lead to a recessively inherited disease called CLN7 disease (late-infantile phenotype, #610951). To date, more than 35 mutations in the MFSD8 gene have been reported, suggesting a complete loss of function of the protein due to the similar clinical phenotype (Aiello et al., 2009; Aldahmesh et al., 2009; Kousi et al., 2009, 2012; Mandel et al., 2014; Mole and Cotman, 2015; Patino et al., 2014; Siintola et al., 2007; Stogmann et al., 2009). The first symptoms of CLN7 disease, which are usually epileptic seizures, occur between 1.5 and 7 years of age. As the disease progress, patients progressively suffer from seizures, loss of speech and vision, ataxia, and involuntary muscle twitching (myoclonias), ultimately ending in premature death between 6.5 and 18 years of age (Kousi et al., 2009; Topcu et al., 2004). Thus, CLN7 disease is characterized by a severe central phenotype and is so far incurable.

Our experiments have shown for the first time that SARS-CoV-2 viral load was 10-fold lower in CLN7-deficient HEK293T cells than in corresponding wild-type cells. The aim of this work was to find a mechanistic model for this observation and to establish a possible link between CLN7 and SARS-CoV-2. This is important not only for understanding the infection cycle of SARS-CoV-2 but also regarding new pharmacological targets for the therapy of COVID-19. In addition, this study provides new insights into the physiological function of CLN7.

## Results

### Infection of CLN7-deficient cells with SARS-CoV-2

**Figure 1:**
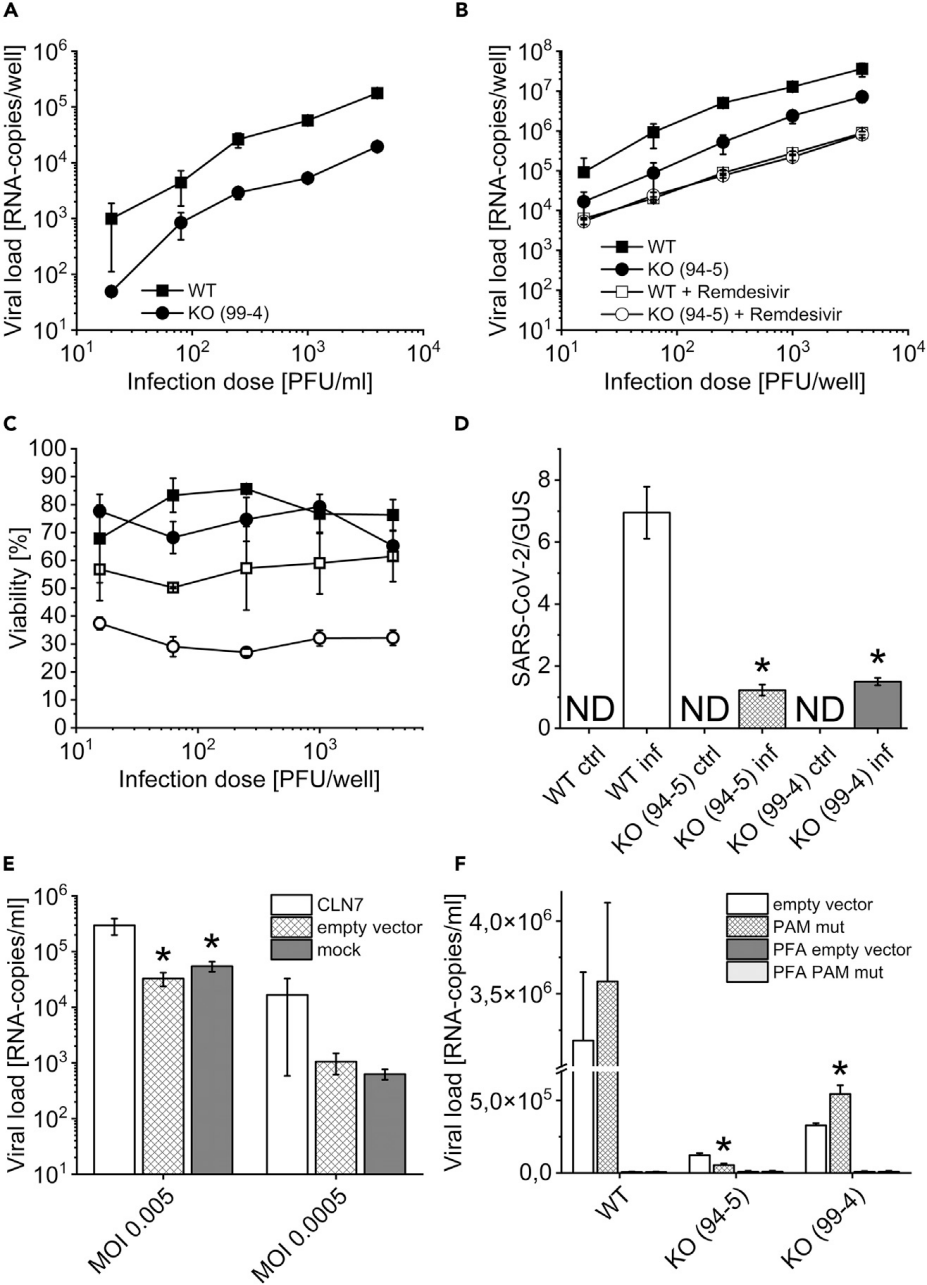
Infection of CLN7-deficient cells with SARS-CoV-2 (A) Infection with SARS-CoV-2 for approximately 48 h revealed that CLN7 knockout cells (line 99-4) had a 10-fold reduction in viral load compared to HEK293T wild-type cells. (B) This experiment was repeated on a second CLN7 knockout cell line (line 94-5) generated with a different guide RNA (CRISPR/Cas9). The data suggest that the decreased viral load was not due to a clonal or off-target effect. Treatment of the cells with Remdesivir further lowered the viral load, and to about the same extent in both experimental groups. (C) Same legend as in b). After infection CLN7 wt and ko cells showed similar viability. However, treatment with Remdesivir was significantly more toxic to knockout cells. (D) CLN7 wild-type and knockout cells (line 99-4 and 94-5) were infected with SARS-CoV-2 for only a short period of time (inf). During this period, the virus can enter the cell but cannot replicate significantly. After trypsinization of the cells, the viral load in the cell lysates was measured and normalized for the housekeeping β-glucuronidase (GUS). Non-infected cells served as a control group (ctrl). As a result, wild-type cells had a viral load about 5 times higher than the two knockout cell lines. This finding suggests that virus uptake may be reduced in CLN7-deficient cells. ∗ Significant compared to wild-type cells. (E) HEK293T wild-type cells were transiently transfected with CLN7 (CLN7), infected with SARS-CoV-2 for 48 h, and subsequently viral load was determined. Sham-transfected cells (mock) and cells with empty vector were used as controls. The virus was added in two concentrations (MOI, MOI). At an MOI of 0.005, overexpression of CLN7 resulted in an almost 10-fold increase in viral load. (F) Rescue experiments with HEK293T wt and ko cells overexpressing CLN7. Since the CRISPR/Cas9 system is consistently active in these cells, a vector was generated in which the PAM sequence was mutated for one of the knockout cell lines (99-4). Here, overexpression of the PAM mutant (PAM mut) showed a 65% greater viral load compared to the empty vector. Overexpression in cell line 94-5, on the other hand, resulted in a 45% lower viral load. d, e: ∗ Significant compared to CLN7-transfected cells. f: ∗ Significant compared to overexpression of empty vector.

In a first experiment, we investigated whether HEK293T CLN7 knockout cells (CLN7 ko 99-4) could be infected with SARS-CoV-2 and whether there were any differences in viral load compared to wild-type cells (CLN7 wt). The cells were incubated with the virus for 48 h. Subsequently, viral load was measured by qPCR in culture medium. It turned out that RNA copy number was reduced by approximately a factor of 10 in knockout cells, regardless of the amount of viruses used for infection (infectious dose, PFU/ml) (Figure 1A). To rule out any off-target effects of the CRISPR/Cas9 system, the experiment was repeated with our second CLN7 knockout cell line generated with a different probe (guide RNA) (CLN7 ko 94-5). Again, the viral load was decreased by 90% (Figure 1B).

In addition, the drug Remdesivir was also tested, which is an approved virostatic agent against SARS-CoV-2. As a result, the viral load in the supernatant was even lower compared to CLN7 ko cells, and no difference was observed between wild-type and knockout cells. To exclude the possibility that these results were the result of increased cell death in the knockout cells, cell viability was also tested (Figure 1C). However, the viability was approximately 70%–80% with no apparent difference between CLN7 wt and CLN7 ko 94-5 cells. Looking at the viability after treatment of the cells with Remdesivir, large differences were observed. While the virostatic agent led to a viability of 50%–60% in wild-type cells, this decreased to a value of only 30%–40% in knockout cells. Remdesivir thus appears to be significantly more toxic for CLN7 knockout cells than for normal HEK293T cells.

### Quantification of virus uptake in CLN7-deficient cells

The decreased viral load in CLN7-deficient cells may have several causes. For example, virus uptake, replication, or exit may be affected. In the following experiment, we investigated whether the entry of SARS-CoV-2 into the cell was decreased (Figure 1D). For this purpose, CLN7 wt cells and both knockout cell lines (line 99-4 and 94-5) were infected for only 12 h. During this time, the viruses can enter the cell but cannot replicate significantly. After harvesting, cells were treated twice with trypsin to remove virus particles adhering to the cell membrane. Subsequently, the cells were lysed and virus RNA was measured by qPCR in the cell lysate. It turned out that wild-type cells (WT inf) had approximately 5 times the viral load compared to the two knockout cell lines (KO (99-4) inf and KO (94-5) inf). As expected, no viral RNA could be found in uninfected cells (ctrl) (ND = not detectable). Assuming that treatment with trypsin indeed removed all virus, these results strongly suggest that uptake of SARS-CoV-2 is reduced in CLN7-deficient cells.

### Quantification of viral load in cells overexpressing CLN7

After the absence of CLN7 led to a reduced viral load in our cell model, the next experiment was to investigate the influence of overexpression (Figure 1E). For this purpose, HEK293T wild-type cells were transiently transfected with CLN7, infected with SARS-CoV-2 for 48 h, and subsequently the viral load was measured. For the infection, an IRES plasmid was used that expresses EGFP in addition to CLN7 (CLN7). In this way, the transfection efficiency could be estimated, which was about 25%. Sham-transfected cells (mock) and cells transfected with the empty plasmid (empty vector) were used as controls. Two different virus concentrations were used for infection, a very low one (MOI 0.0005) and a moderately high one (MOI 0.005). The MOI indicates the numerical ratio of infectious virus particles to target cells. As a result, at a MOI of 0.005, overexpression of CLN7 had increased viral load by almost 90%. Although this trend was also evident at an MOI of 0.0005, the scattering was particularly large in cells transfected with CLN7. In comparison, cells transfected with empty vector and sham-transfected cells behaved very similarly.

### Effect of CLN7 rescue on SARS-CoV-2 viral load

Since the CRISPR/Cas9 system is permanently active in our HEK293T CLN7 knockout cells, the expression vector had to be modified to perform rescue experiments. For one of the two knockout cell lines (99-4), the PAM (protospacer adjacent motif) sequence was mutated, which serves as a recognition or tag sequence for the CRISPR/Cas9 system. Thus, overexpression of this vector should lead to a rescue in cell line 99-4, whereas it should have no effect in cell line 94-5. The data are shown in Figure 1F. In addition to the overexpression in wild-type cells (WT) and both knockout cell lines (94-5 and 99-4), PFA controls are also shown. These controls allow us to estimate what proportion of the measured viral load is not caused by replication but by adhesion and by nonspecific uptake of virus particles. It thus shows the expected nonspecific background. A significantly higher viral load in non-fixed cells indicates that the virus can replicate in the cells.

Again, the difference between wild-type and knockout cells was very obvious (empty vector) and even more pronounced here than in the previous experiments. In addition, the PFA controls show that HEK293T cells can be infected. While overexpression of the vector with mutated PAM sequence led to a 65% increase in viral load in cell line 99-4 (compared to empty vector!), it led to about 55% reduction in cell line 94-5. However, the values of wild-type cells were not reached by far.

### Quantification of GM1 expression in CLN7 knockout cells and patient fibroblasts

**Figure 2:**
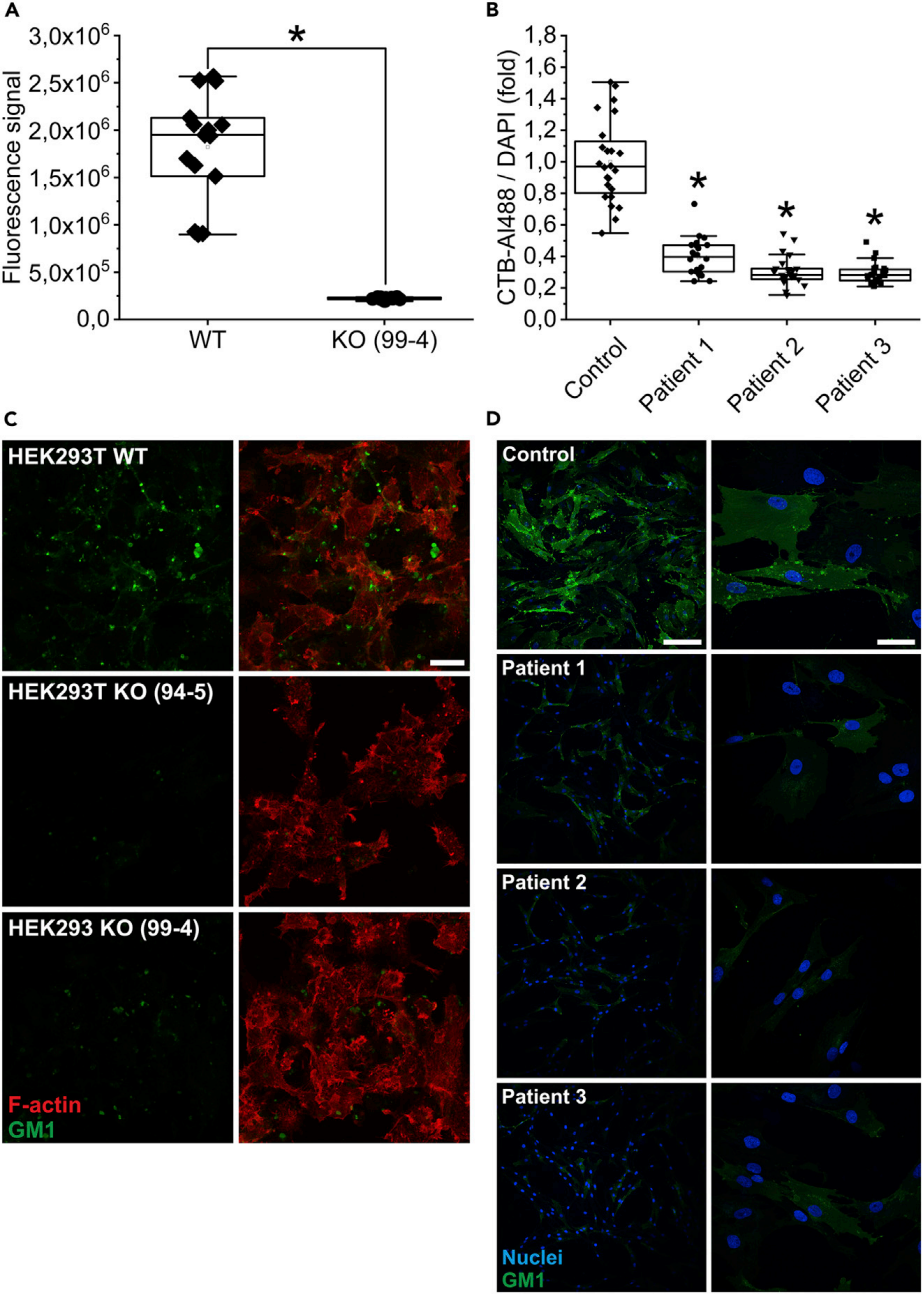
GM1 measurement in CLN7 knockout cells and patient fibroblasts (A) Flow cytometric quantification of GM1 expression in HEK293T wt and ko cells (line 99-4) using fluorescence-coupled cholera toxin, subunit B. CLN7-deficient cells exhibited a 87% reduction in GM1 expression. (B) GM1 measurement in fibroblasts from a healthy individual (Control) and from three patients with CLN7. Here, quantification was performed using a fluorescence microscope. Consistent with the experiment on HEK293T cells, cells from patients exhibited a 60% reduction in GM1 expression. ∗ Significant compared to wild-type cells in a) and compared to fibroblasts from a healthy individual in b). (C) GM1 staining (green) on fixed CLN7 wt (top row) and ko cells (line 94-5 middle row, line 99-4 bottom row). F-actin was also stained (red) to visualize the cells. The last image of a row shows the merged image. Microscopic evaluation of the cells also showed that CLN7 ko cells expressed significantly less GM1 in their cell membrane. Scale bar: 20 μm. (D) GM1 staining (green) of human fibroblasts from a healthy individual (top row) and from three patients with CLN7 (bottom three rows). As in the HEK293T cell model, GM1 expression was reduced by approximately 33% in patients. Nuclei are shown in blue. Scale bars of left column: 200 μm, right column: 50 μm.

In this experiment, the expression of the ganglioside GM1 in the cell membrane of CLN7 wt and ko cells was quantified by flow cytometry. Since this ganglioside is preferentially localized in lipid rafts, it can also be used as a marker for these specialized membrane regions. Fluorescently labeled cholera toxin (subunit B), which binds specifically to GM1, was used for staining. The experiment had shown that GM1 was decreased by approximately 87% in CLN7 knockout cells (line 99-4) (Figure 2A). Figure 2C shows microscopic images of a GM1 staining (green) on fixed cells. F-actin (red) was also stained to visualize the cells. Consistent with the flow cytometry experiments, GM1 expression was significantly decreased in the two knockout cell lines (line 94-5: middle row and line 99-4: bottom row) compared to wild-type cells (top panel).

To exclude that this phenomenon occurs only in HEK293T cells, the experiment was also repeated using fibroblasts of patient with CLN7. Skin biopsies from three patients with a very severe course of CLN7 disease were examined. A healthy person of approximately the same age served as a control. Quantification was also performed using fluorescently labeled cholera toxin, subunit B. However, mean fluorescence intensity was determined by microscopic images of fixed cells. Consistent with our CLN7-deficient HEK293T cell model, an approximately 60% reduction in GM1 expression was observed in patient’s fibroblasts compared with healthy subjects (Figure 2B). Microscopic images of a GM1 staining of these fibroblasts are shown in Figure 2D. Significantly less GM1 signal was observed in the cell membrane of patients with CLN7 (second row: patient 1, third row: patient 2, fourth row: patient 3) compared to fibroblasts from a healthy individual (top row).

### Quantification of macropinocytosis activity in CLN7 knockout cells

**Figure 3:**
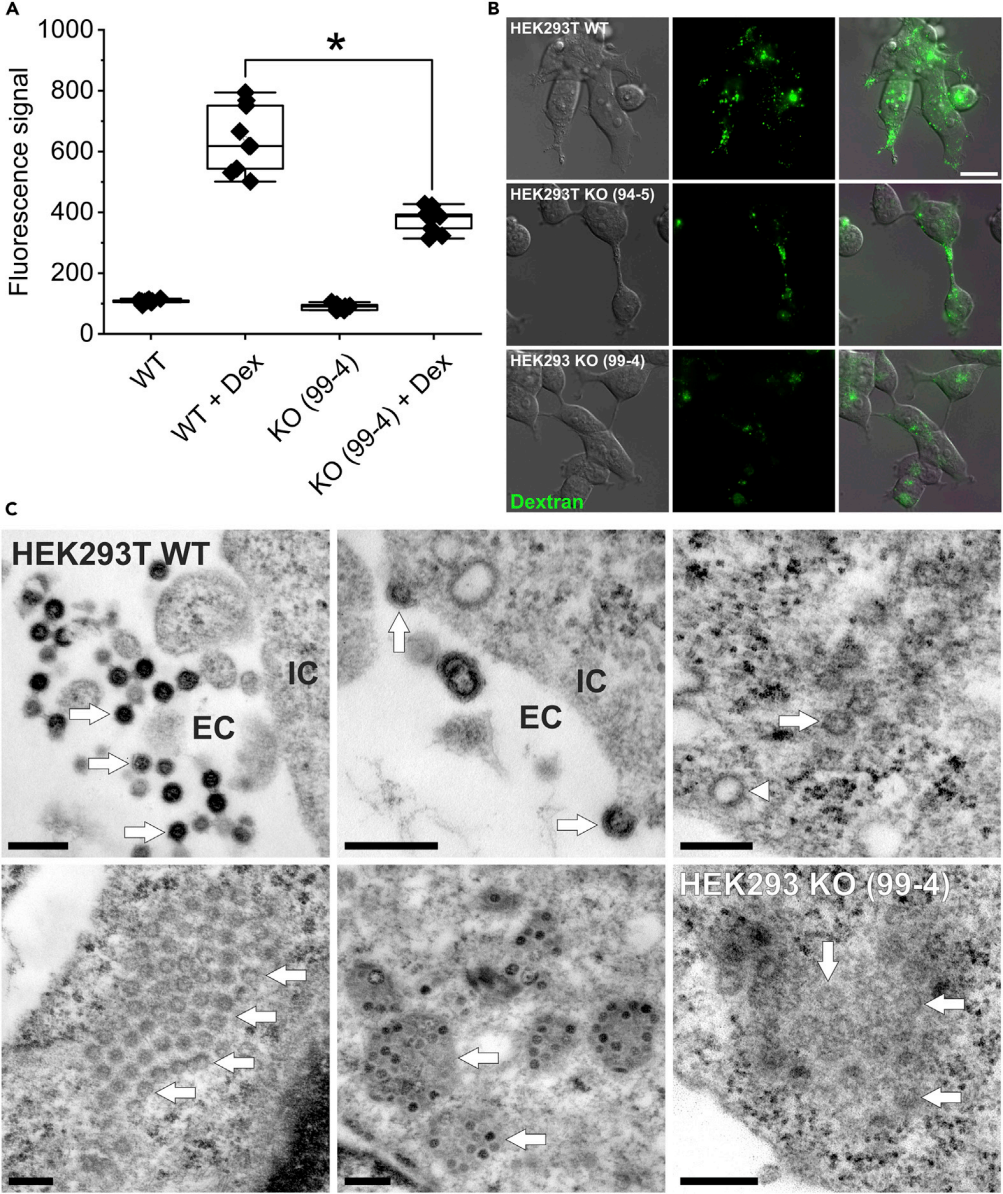
Measurement of macropinocytosis and electron microscopic studies (A) Fluorescence-coupled dextran (10,000 kDa) was used as a marker for macropinocytosis. Quantification was performed by flow cytometry. CLN7 knockout cells (KO (99-4) + Dex) exhibited a 41% reduced macropinocytosis rate compared with wild-type cells (WT + Dex). In contrast, the autofluorescence of the cells at 647 nm was the same (WT and KO (99-4)). Macropinocytosis is discussed as a mechanism for uptake of SARS-CoV-2 into cells. ∗ Significant compared to wild-type cells. (B) Comparing fluorescence images (top row: WT, middle row: KO 94-5, bottom row: KO99-4), a reduced number of macropinosomes (green) can be observed in both knockout cell lines. Scale bar: 20 μm. (C) Electron microscopic studies. Top left: SARS-CoV-2 particles are in the process of infecting a HEK293T cell. The viruses (three exemplary arrows) are located in the extracellular space (EC). The cell is cropped in the right part of the image (IC = intracellular space). As described in literature, the particles are between 80 and 120 nm in size. Top center: viruses which are about to enter the cell (arrows). Top right: Intracellularly, the viruses always appear in groups (arrow points to a virus particle as an example). They can be easily confused with clathrin-coated vesicles (arrowhead), which have a similar size. Bottom left: A group of viral particles in the cytosol (arrows). The ERGIC membrane envelope is not visible with this method of contrast. Bottom center: In some images, the contents of ERGIC vesicles as well as the viruses themselves are clearly more electron dense (two exemplary arrows), possibly indicating a different stage of maturation. Bottom right: viruses were also found in CLN7-deficient cells, but at a much lower abundance. Scale bar: 250 nm.

In a next set of experiments, macropinocytosis activity in HEK293T CLN7 wt and ko cells was examined by flow cytometry. For this purpose, cells were incubated with fluorescence-coupled dextran (10,000 kDa), which can only enter the cell via macropinocytosis. As a result, macropinocytosis rate in CLN7 knockout cells was reduced by approximately 41% compared with wild-type cells (Figure 3A). This diminished macropinocytosis activity could be directly related to the reduced GM1 expression in CLN7 knockout cells, as GM1 is an important component of lipid rafts and lipid rafts in turn appear to be important for macropinocytosis processes (“lipid raft-dependent macropinocytosis”) (Imelli et al., 2004; Sonnino et al., 2007)).

When viewing the dextran-loaded cells under the fluorescence microscope (Figure 3B), it was apparent that both CLN7-deficient cell lines (middle row: line 94-5, bottom row: line 99-4) contained significantly less dextran-positive vesicles than corresponding wild-type cells (top row).

### Electron microscopy of SARS-CoV-2-infected CLN7 wild-type and knockout cells

In addition to quantifying SARS-CoV-2 particles, we also attempted to visualize them by electron microscopy in CLN7 ko and wt cells. In Figure 3C, the top row and the first two pictures of the second row show HEK293T wt cells infected with the virus for 48 h. We could clearly identify virus particles in these cells, indicating that our cell model can be infected with SARS-CoV-2. As it is shown by picture one and two in the second row, the virus particles in the cytosol always occur in groups. This is because several particles are always sequestered from the ERGIC in a common membrane envelope. Thus, the viruses are never present individually in the cytosol. When single viruses are identified, they are usually clathrin-coated vesicles of similar size (top row, last picture, arrowhead) (Brahim Belhaouari et al., 2020; Goldsmith et al., 2020; Miller and Goldsmith, 2020).

In contrast to wild-type cells, it was enormously tedious to find virus particles in CLN7 knockout cells. The last picture of the second row shows one of two images where virus particles were identified in these cells. The very rare occurrence suggests that uptake of the virus into the cell or replication is impaired.

### Cell migration and cell adhesion in CLN7 knockout cells

**Figure 4:**
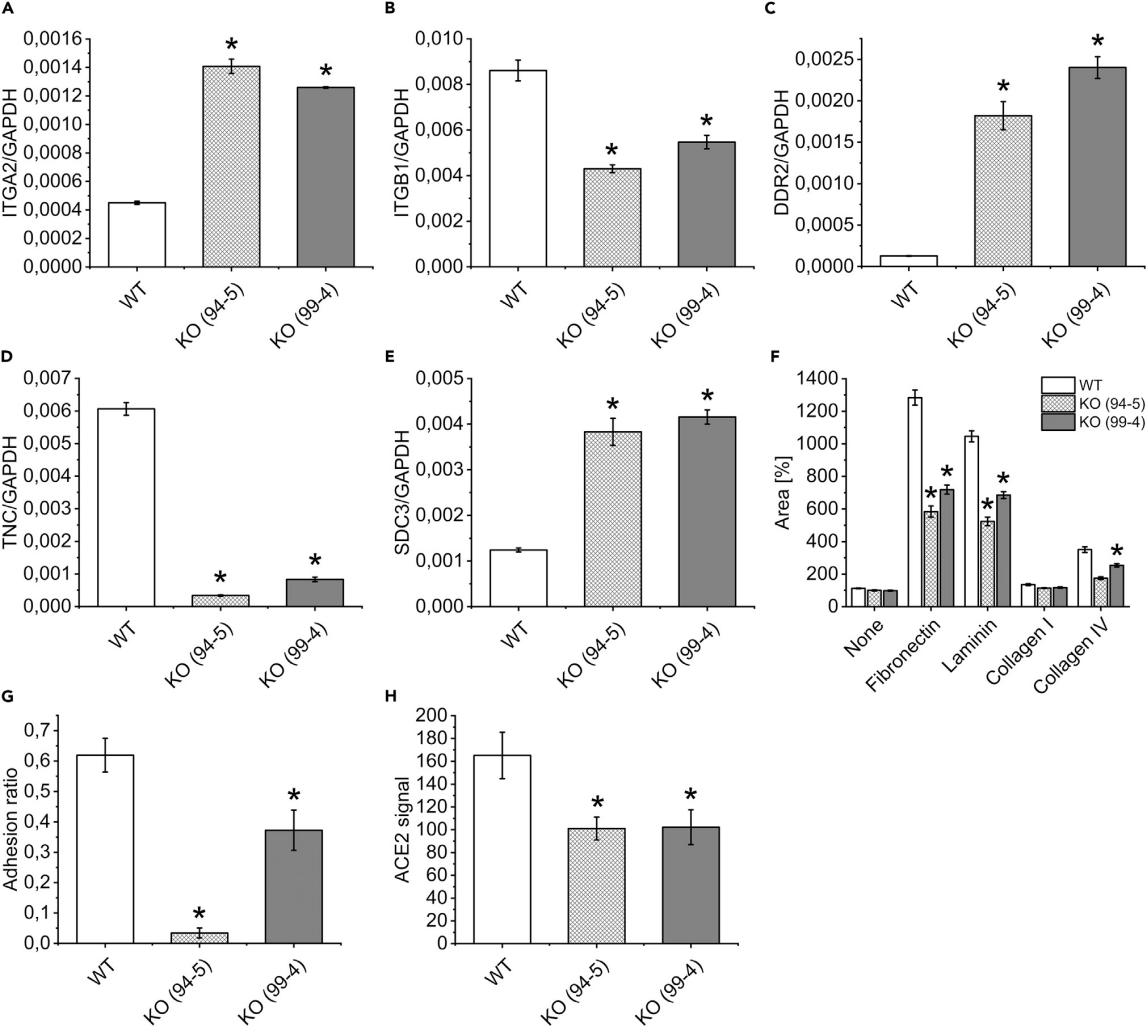
Measurement of cell migration, adhesion, and ACE2 surface expression in CLN7-deficient cells (A–E) Quantitative PCR for proteins involved in cell migration and adhesion. CLN7 wt and both knockout cell lines (99-4 and 95-5) were measured. In addition to integrin α2 (ITGA2) and β1 (ITGB1), discoidin domain-containing receptor 2 (DDR2), tenascin C (TNC), and syndecan 3 (SDC3) were found to be regulated. Levels were normalized to GAPDH expression. (F) Measurement of cell migration: cells were cultured in the form of “neurospheres” and then transferred to differently coated supports for outgrowth. The area after 24 h of growth was quantified relative to the original area of the neurospheres (in %). Notably, on fibronectin and laminin, CLN7 knockout cells grew significantly worse, suggesting altered integrin expression. (G) Measurement of cell adhesion: cells were cultured in fibronectin-coated ibidi flow chambers and exposed to a flow rate of 50 mL/min for 5 min. Shown is the adhesion quotient, which is calculated from the nuclear area after divided by the nuclear area before shear stress. The closer this value is to one, the higher the adhesion of the cells. CLN7-deficient cells show significantly reduced adhesion ability compared to wild-type cells. (H) Quantification of ACE2 surface expression by flow cytometry. Compared to wild-type cells, both CLN7 ko cell lines (99-4 and 94-5) showed reduced ACE2 expression of about 40%. Both, integrins and ACE2 are thought to serve as receptors for SARS-CoV-2 attachment to target cells. ∗ Significant compared to wild-type cells.

During routine work on CLN7 knockout cells, we observed that their growth and detachment behavior differed from wild-type cells. Therefore, mRNA expression of some proteins important for cell migration and adhesion was examined by qPCR (Figures 4A–4E). As a result, two proteins of the integrin family were differentially regulated in CLN7 ko cells, namely integrin α2, for which mRNA expression was approximately 3-fold greater in ko cells (Figure 4A) and integrin β1, which was reduced by 50%–60% in ko cells (Figure 4B). Differential regulation was also observed for discoidin domain-containing receptor 2 (DDR2, Figure 4C), tenascin C (TNC, Figure 4D), and syndecan 3 (SDC3, Figure 4E), all of which have been reported to be involved in cell migration and adhesion.

To investigate the relevance of these data, the migratory ability of CLN7 wt and ko cells was compared (Figure 4F). For this purpose, cells were first seeded on agarose. The lack of adhesion caused the cells to assemble in the form of a sphere (also called “neuropheres”). These neurospheres were then transferred to wells coated with different coating media (no coating, fibronectin, laminin, and collagen I and IV). After 24 h, the area of outgrowing cells was quantified and related to the area of the original cell cluster (area in % of the original area). This experiment revealed that CLN7 wild-type cells grew out significantly better than the two knockout cell lines, especially on fibronectin and laminin, but also to a lesser extent on collagen IV.

In addition to migration ability, the adhesion of CLN7 ko cells was also investigated (Figure 4G). Based on the data from the migration experiment, fibronectin was chosen as the coating. Cells were cultured in ibidi flow chambers for this experiment. Nuclear area, which is proportional to cell number, was used to quantify the cells. A roller pump was used to expose the cells to a strong flow for 5 min. Before as well as after applying the flow, an image of the nuclei was taken, which were stained with a fluorescent dye. Both images were from the same location. By dividing the core area after by the core area before application of the flow, the adhesion quotient is obtained, which is shown in Figure 4G. The smaller this value, the more poorly the cells adhere to the support. In agreement with the cell migration experiment, both knockout cell lines show significantly reduced adhesion compared to wild-type cells. This experiment also suggests that the absence of CLN7 is associated with an altered integrin expression.

### Quantification of ACE2 surface expression in CLN7 knockout cells

Because the ACE2 receptor appears to be crucial for SARS-CoV-2 virus uptake, we examined ACE2 surface expression in CLN7-deficient cells by flow cytometry (Figure 4H). A polyclonal antibody against ACE2 was used. Because the signal was quite weak due to a low receptor density, an additional negative control was performed with an isotype control antibody. Isotype controls are designed to reveal the extent of background signal from the primary antibody. In the data shown, the fluorescent signal from the isotype control antibody was subtracted from the signal from the ACE2 antibody. In both CLN7 knockout cell lines, an approximately 40% reduction in ACE2 surface expression was observed. The reasons for this reduced expression in the absence of CLN7 are unclear but may be indirectly related to reduced GM1 expression or impaired lipid raft function. Because the ACE2 receptor resides preferentially in these membrane domains (Lu et al., 2008), a lack of functional lipid rafts could have a direct effect on ACE2 surface expression.

## Discussion

We observed in a CLN7-deficient HEK293T cell line that the absence of CLN7 resulted in significantly weaker infection with SARS-CoV-2. The aim of this study was to investigate a possible link between CLN7 and SARS-CoV-2. To minimize clonal effects or off-target effects in cells generated with CRISPR/Cas9, most experiments were performed on two different knockout cell lines (94-5 and 99-4) for which different guide RNAs were used. In both cell lines, infection with SARS-CoV-2 resulted in approximately 90% reduced viral load compared with wild-type cells (Figures 1A and 1B). It is very unlikely that this is the result of an increased mortality of knockout cells, as they exhibited the same viability as wild-type cells after the infection (Figure 1C). In comparison, treatment with the approved SARS-CoV-2 antiviral drug Remdesivir exhibited a further reduction in viral load of about 90%. However, wild-type and knockout cells were affected to the same extent (Figure 1B). A measurement of cell viability also showed that Remdesivir appeared to be significantly more toxic to CLN7 knockout cells than to wild-type cells (Figure 1C). This should be considered with regard to antiviral therapy in patients with CLN7.

The reduced viral load in CLN7-deficient cells can have various causes. For example, it is conceivable that adherence or entering of the viruses into the target cell is reduced. Furthermore, virus replication or budding may be impaired. To test this hypothesis, an experiment was performed in which the cells were incubated with the virus for only a very short time (12 h) (Figure 1D). During this time, the viruses can enter the cell but cannot replicate significantly. To get rid of the input virus, attempts were made to detach all adherent virus particles on the outside of the cell membrane using trypsin. In comparison to the previous experiments, the viral load was quantified in the cell lysate rather than in the cell culture medium. The experiment showed that wild-type cells contained approximately five times as much viral nucleic acids as the two CLN7 knockout cell lines. Thus, in the case where all adherent viruses could be completely removed, it appears that SARS-CoV-2 penetration into target cells is impeded. In addition, if the virus particles could not be completely removed, this may mean that the attachment of the particles to the cells is diminished. In any case, it seems to be the initial steps of the infection process that are affected in CLN7-deficient cells. This is also supported by the electron micrographs of SARS-CoV-2-infected cells (Figure 3C). While infected cells were regularly found in HEK293T wild-type cells, it was much more difficult to find infected cells in both of the knockout cell lines. If virus exocytosis had been impaired, one would have expected cells that contained significantly more virus than the wild-type cells. However, considering only the electron micrographs, it cannot be excluded that virus replication is slower in the knockout cells.

In further experiments, we could show that overexpression of CLN7 led to a significant increase in viral load, i.e. it had exactly the opposite effect of a CLN7 knockout. This effect was particularly evident at an MOI of 0.005 and was only apparent as a trend at the low MOI of 0.0005. It may be that at such low concentrations, small variations in experimental conditions ended up having a large effect, which is why the scatter was particularly high here. However, at an MOI of 0.005, viral load was 5.4-fold higher in CLN7-expressing cells than in sham-transfected cells and 9-fold higher than in cells with empty vector. Co-expressed EGFP was used to estimate the transfection efficiency, which was approximately 25%. Despite this low transfection rate, there was a clear effect on viral load.

An important factor for SARS-CoV-2 infection seems to be the presence of lipid rafts (Li et al., 2021; Sviridov et al., 2020). Lipid rafts are specialized membrane domains rich in sphingolipids and cholesterol. Certain proteins are thought to preferentially reside in these areas, concentrating in a confined space (Simons and Ikonen, 1997). In addition to the biological roles of lipid rafts, there is much evidence that they are important for the entry, assembly, and budding of pathogenic microorganisms (Kovbasnjuk et al., 2001; Rawat et al., 2003; Suomalainen, 2002). For human HIV-1 (Popik et al., 2002) and for SARS-CoV (Lu et al., 2008), lipid rafts have been shown to play an important role in viral entry into cells. Lipid rafts are thought to be responsible for the enrichment of the ACE2 receptor in confined membrane areas, which significantly facilitates the docking of SARS-CoV-1 to target cells. Disruption of lipid rafts reduced SARS-CoV-1 uptake into Vero E6 cells by approximately 90%, although the absolute amount of ACE2 receptors on the cell surface barely changed (Lu et al., 2008).

An essential component of lipid rafts is the ganglioside GM1, a glycosphingolipid found primarily in the outer leaflet of the cell membrane. GM1 affects the function of lipid raft resident proteins (Mahmood et al., 2013) and is thought to play an important role in cell adhesion and migration (Fuentes and Butler, 2012; Guillaume et al., 2013) as well as cell polarity (Bisel et al., 2013). Our measurements revealed that CLN7-deficient HEK293T cells expressed significantly less GM1 in the cell membrane than corresponding wild-type cells. This result was confirmed in fibroblasts from patients with CLN7 (Figure 2). It can be assumed that this reduced GM1 content is associated with reduced lipid raft function. For some viruses, it has been shown that disruption of lipid rafts by cholesterol deprivation (Fujita et al., 2007) resulted in a reduced infection of target cells (Liu et al., 2002; Lu et al., 2008). In addition, the mechanism of action of hydroxychloroquine, which shows good efficacy against SARS-CoV-2 *in vitro*, is thought to result, in part, from the disruption of GM1-containing lipid rafts (Yuan et al., 2020). Recent publications also describe that GM1 is a cofactor for the attachment of SARS-CoV-2 to target cells, i.e., to be directly involved in the mechanism of infection (Fantini et al., 2020a). Therefore, it is conceivable that the reduced viral load of CLN7-deficient cells is directly caused by a reduced GM1 content of the cell membrane and, in addition, indirectly due to impaired lipid raft function.

In rescue experiments (Figure 1F), we attempted to restore viral load in our HEK293 knockout cell model to the level of wild-type cells by overexpressing CLN7. To do this, we had to mutate the PAM sequence in our CLN7 expression vector, since the CRISPR/Cas9 system is permanently active in our HEK293 cells. The mutation was only inserted for the knockout cell line 99-4, which is why the vector should only lead to rescue here. Indeed, we observed a 65% increase in viral load in cell line 99-4 compared to the overexpression of the empty vector. In the knockout cell line 94-5, on the other hand, the viral load had actually decreased by 55%. However, looking at wild-type cells, we find that overexpression of the (empty) vector led to significantly higher viral loads here. It is not clear why the rescue effect was only relatively small. One problem may be that the duration of overexpression of 48 h was too short for a complete rescue. However, longer culturing of HEK293T cells resulted in detachment of the cells, which prevented longer expression times from being performed. If the transfected cells were split and reseeded, then predominantly the non-transfected cells (indicated by the lack of GFP co-expression) grew back. It is conceivable that CLN7 is (indirectly) involved in the export of ganglioside degradation products from lysosomes. However, in CLN7-deficient cells, GM1 appears to be trapped in multilamellar bodies that are part of the ceroid lipofuscin. Thus, it may be that GM1 must first be resynthesized after restoration of CLN7 function and cannot be recycled from the lysosome, or can only be recycled very slowly. Lysosomal recycling of gangliosides appears to be important for the GM1 content of a cell (Ghidoni et al., 1987; Trinchera et al., 1990).

In further experiments, we observed that CLN7 knockout cells had an approximately 41% decreased macropinocytosis activity (Figures 3A and 3B). Macropinocytosis is an important mechanism for the entry of certain viruses into cells (Izumida et al., 2020), which is also discussed for SARS-CoV-1 (Freeman et al., 2014) and SARS-CoV-2 (Glebov, 2020), although the literature is controversial for the latter (Li et al., 2021). While the observed macropinosomes were of comparable size in CLN7 wt and ko cells, their number was reduced in both knockout cell lines. The lower macropinocytosis activity may be the result of decreased GM1 expression, as lipid raft-dependent macropinocytosis is dependent on GM1 expression in the cell membrane (Lajoie and Nabi, 2007; Pang et al., 2004). Overexpression of CLN7 leads to the formation of very large lysosomes in cells (Steenhuis et al., 2010). It can be speculated that this could be the result of an increased pinocytosis rate, or more precise, the result of an imbalance from membrane internalization to membrane retraction.

During experiments on CLN7 knockout cells, it has been noticed that both their growth and adhesion to the bottom of the dishes differed from wild-type cells. To investigate this finding, the expression of certain proteins involved in cell adhesion and migration was examined by qPCR. Integrins α2 (ITGA2) and β1 (ITGB1) were found to be regulated, as well as discoidin domain-containing receptor 2 (DDR2), tenascin C (TNC), and syndecan 3 (SDC3) (Figures 4A–4E). The integrin α2 and β1 heteromer is also known as VLA-2, GPIa-IIa, or CD49b and serves as a receptor for many extracellular matrix proteins (Elices and Hemler, 1989; Kirchhofer et al., 1990; Staatz et al., 1989). The protein DDR2 is an adhesion-promoting protein that is thought to interact with α2β1-integrin, among other proteins, and enhance its adhesion to collagen (Xu et al., 2012). TNC is described as an adhesion-modulating protein because it inhibits cellular adhesion to fibronectin (Chiquet-Ehrismann, 2004). SDC3, on the other hand, is also involved in cell adhesion and migration processes and is thought to play an important role, particularly in the CNS (Kaksonen et al., 2002; Kim et al., 1994). Interestingly, syndecans are also thought to be important in the uptake of SARS-CoV-2 into cells (Hudak et al., 2021). In further experiments, the adhesion and migratory ability of CLN7 knockout cells was quantified. It turned out that CLN7-deficient cells migrate slower, especially on fibronectin and laminin (Figure 4F). In addition, knockout cells adhere worse to the substrate, which was investigated for fibronectin (Figure 4G). These results all indicate that both cell migration and cell adhesion are impaired in CLN7 knockout cells.

Recent data indicate that proteins from the integrin family also contribute to the attachment of SARS-CoV-2 to target cells (Carvacho and Piesche, 2021; Sigrist et al., 2020; Simons et al., 2021). To do this, the virus has an RGD motif (Arg-Gly-Asp) in the spike protein that recognizes integrins. Normally, integrins are important for cell adhesion and migration, but are also used by viruses (e.g., Ebola, Epstein-Barr virus, rotavirus, and human cytomegalovirus) as receptors for entry (Hussein et al., 2015). SARS-CoV-2 is thought to spread significantly faster and more effectively due to the RGD motif than SARS-CoV-1, which lacks such a motif in the spike protein. A recently published paper describes that β1 integrin in particular mediates this interaction and may serve as an alternative, ACE2-independent receptor for the spike protein of SARS-CoV-2 (Park et al., 2021). In our CLN7 knockout cells, β1 integrin mRNA is decreased by approximately half, which is most likely associated with decreased protein expression based on the adhesion and migration experiments. Thus, decreased β1 integrin expression could also contribute to the poorer ability of CLN7-deficient cells to become infected with SARS-CoV-2.

In another experiment, ACE2 surface expression was quantified in CLN7 knockout cells by flow cytometry (Figure 4H). An approximately 40% reduction was observed in both knockout cell lines compared with wild-type cells, which may also contribute to the low viral load in CLN7-deficient cells. It is unclear how a lack of CLN7 protein expression may affect the expression level of ACE2. However, it is conceivable that this occurs indirectly through reduced lipid raft function. Indeed, ACE2 colocalizes extensively with GM1 and thus preferentially resides in lipid rafts. The narrow space in which the receptors are concentrated makes a successful interaction with the spike protein more likely (Sorice et al., 2020). It is also worth noting that β1 integrin is also localized in lipid rafts (Runz et al., 2008; Wang et al., 2010). Thus, altered composition of lipid rafts in CLN7 knockout cells could indirectly influence the expression levels of proteins that preferentially incorporate into these structures.

It should be mentioned that the reduced viral load of CLN7-deficient cells may also be the consequence of impaired lysosome function in general. This can affect membrane trafficking as well as lysosomal pH. Recently, a publication showed that SARS-CoV-2 entry into HEK293T cells depends on an acidic lysosomal pH (Li et al., 2021). This appears to be the result of, among other things, decreased function of cathepsins, which are dependent on acidic pH (Blaess et al., 2020; Pislar et al., 2020). Cathepsin L, in particular, appears to play a prominent role in the entry of SARS-CoV-2 into the cell and is therefore discussed as a pharmaceutical target against COVID-19 (Zhao et al., 2021). It is known that lysosomal pH is elevated in many forms of neuronal ceroid-lipofuscinosis. This is also true in CLN7 disease (Holopainen et al., 2001; Wang et al., 2021). Thus, it is conceivable that at least part of the reduced viral load of CLN7-deficient cells is indirectly caused by nonspecific lysosome damage. However, considering the fact that overexpression of CLN7 leads to a significantly increased viral load, it is very unlikely that lysosome damage alone is responsible for the observed effects.

In summary, CLN7 knockout cells are more resistant against an infection with SARS-CoV-2. We hypothesize that this is the result of decreased GM1 expression in CLN7 knockout cells and an associated dysfunction of lipid rafts. CLN7 thus appears to be another protein that directly or indirectly influences the infection process. Currently, there are only a few specific and effective drugs that positively affect the clinical course of COVID-19 when used early. Our results indicate that CLN7 might be a potential pharmacological target. In addition, this study provides important insights into the physiological function of CLN7. Considering the severely reduced GM1 expression in CLN7-deficient cells, it is conceivable that CLN7 disease could actually be a GM1 gangliosidosis. Both diseases are characterized by a severe central phenotype. In the case of CLN7 disease, GM1 deficiency could be a cause of the problem, as this ganglioside is thought to be particularly important for neuronal function (Ichikawa et al., 2009; Sandhoff and Harzer, 2013). GM1 may also be suitable as a biomarker for rapid diagnosis or differentiation of CLN7 disease.

### Limitations of the study

The vast majority of the data were generated with only one virus strain (“CA”). This somewhat limits the significance of the study. Different SARS-CoV-2 variants use different strategies to enter the cell (Halfmann et al., 2022). While some variants predominantly use TMPRSS2 to fuse with the cell membrane (e.g., Delta), there are other variants that enter cells primarily via endocytosis (e.g., Omicron). Thus, it is not clear whether CLN7 knockout also leads to reduced viral load using other viral variants. This is the subject of further research.

In addition, it is not certain whether CLN7, as a lysosomal protein, directly interacts with the virus at all. If not, the data would most likely be the result of an indirect effect, such as decreased lysosomal function. However, two findings argue against this. First, it is now well established that SARS-CoV-2 infects HEK 293T cells predominantly via endocytosis (Bayati et al., 2021; Ou et al., 2021). The endosomes in which the viruses initially reside fuse with lysosomes in a later process, bringing CLN7 into direct contact with the virus. Second, a recent study has shown that approximately 22% of cellular CLN7 is localized in the plasma membrane (Steenhuis et al., 2010). Even if this is only an intermediate stop for the protein on its way into the lysosomal membrane, as postulated in this study, this still leads to a direct interaction between SARS-CoV-2 and CLN7.

## STAR★Methods

### Key resources table

**Table:** 

REAGENT or RESOURCE	SOURCE	IDENTIFIER
**Antibodies**		

Human ACE-2 APC-conjugated Antibody	R&D Systems	Cat#FAB933A
Goat IgG APC-conjugated Antibody	R&D Systems	Cat#IC108A

**Bacterial and virus strains**		

SARS-CoV-2 strain CA (spike mutations with reference to Wuhan-Hu-1: D218E, P323L, R203K, G204R, D614G)	Institute of Clinical Microbiology and Hygiene, University Hospital Regensburg	Clinical SARS-CoV-2 isolate, GenBank accession number: MZ675816
DH5α Competent Cells	Thermo Fisher Scientific Inc. (US)	18,263,012

**Chemicals, peptides, and recombinant proteins**		

β-glucuronidase	Merck KGaA (DE)	Cat#3707580001
Alexa Fluor 647nm-coupled Cholera Toxin Subunit B	Thermo Fisher Scientific Inc. (US)	
Alexa Fluor 488nm-coupled Cholera Toxin Subunit B	Thermo Fisher Scientific Inc. (US)	Cat#C34775
Accutase Cell Detachment Solution	Capricorn Scientific GmbH (DE)	Cat#ACC-1B
Phalloidin-AF647	Life Technologies GmbH (DE)	Cat#A22287
Fetal Calf Serum (FCS)	Capricorn Scientific GmbH (DE)	Cat#FCS-62A
Paraformaldehyde	Merck KGaA (DE)	Cat#158127
DAPI (HOECHST-Straining-Solution - Bis-Benzimide H33258)	Sigma-Aldrich (US)	Cat # 94,403-1ML
DAPI Fluoromount-G	SouthernBiotech	Cat# 0100-20
Dextran (10 kDa) coupled to Alexa Fluor 546 nm	Thermo Fisher Scientific Inc. (US)	Cat#D22911
Dextran (10 kDa) coupled to Alexa Fluor 647 nm	Thermo Fisher Scientific Inc. (US)	Cat#D22914
Trypsin	Capricorn Scientific GmbH (DE)	Cat#TRY-2B10
Ethylendiamintetraacetat (EDTA)	Merck KGaA (DE)	Cat#1084540100
Agarose, universal (peqGold)	VWR International (US)	Cat#PEQL35-1030AL
Cultrex Mouse Laminin I	R&D Systems/Bio Techne GmbH	Cat # 3400-010-02
Bovine Fibronectin	PromoCell	Cat#C-43060
Collagen I from calf-skin	Sigma-Aldrich (DE)	Cat#C8919
Cultrex Mouse Collagen IV	R&D Systems/Bio Techne GmbH	Cat#3410-010-02
Penicillin Streptomycin (10.000 U/ml)	Capricorn Scientific GmbH (DE)	Cat#PS-B (100 mL)
bisBenzimide H 33,258 (HOE 33258)	Merck (DE)	Cat#B2883-100MG
Glutaraldehyde 25%, solution in water	SERVA Electrophoresis GmbH (DE)	Cat#23114.02
Cacodylic acid sodium salt	Carl Roth GmbH + Co. KG (DE)	Cat#5169.2
Osmium tetroxide 4%, aqueous solution	Electron Microscopy Science (US)	Cat#19150
Epoxy embedding medium	Sigma-Aldrich (DE)	Cat#45345-1L-F
Epoxy embedding medium, hardener DDSA	Sigma-Aldrich (DE)	Cat#45346-250ML-F
Epoxy embedding medium, hardener MNA	Sigma-Aldrich (DE)	Cat#45347-1L-F
Glycidether Accelerator DMP-30	Carl Roth GmbH + Co. KG (DE)	Cat#8621.1
Uranyl acetate dihydrate	Merck (DE)	Cat#6159-44-0
Lipofectamine™ 3000	Thermo Fisher Scientific Inc. (US)	Cat#L3000008
X-TremeGene 9 Transfection Reagent	Sigma-Aldrich (US)	Cat# 06,365 779,001
Polybrene	EMD Millipore (US)	Cat#TR-1003-G
Puromycin	Sigma-Aldrich (US)	Cat#P8833
Alexa Fluor™ 532 Phalloidin F-actin	Thermo Fisher Scientific Inc. (US)	Cat# A22282
RNase inhibitor	Applied Biosystems, Darmstadt (DE)	Cat# N8080119
Taq-Path-Mix	Metabion international, Planegg (DE)	N/A
IGEPAL CA-630	VWR International, Radnor (US-PA)	Cat# J61055.AP
Remdesivir	Gilead Sciences	Pharmacy of the University Hospital Regensburg

**Critical commercial assays**		

NucleoSpin® RNA Kit	Macherey-Nagel GmbH & Co. KG (DE)	Cat#REF740955.250
Reverse Transcription System	Promega Corporation (US)	Cat#A3500
Takyon™ No Rox SYBR® MasterMix dTTP Blue	Eurogentec (BE)	Cat# UF-NSMT-B0701
HiYield® PCR Clean-up/Gel Extraction Kit	Süd-Laborbedarf GmbH (DE)	Cat#30 HYDF100
HiYield® Plasmid Mini Kit	Süd-Laborbedarf GmbH (DE)	Cat#30 HYPD100
NucleoBond® Xtra Midi Plasmid purification Kit	Macherey-Nagel GmbH & Co. KG (DE)	Cat#REF740410.50
NucleoSpin® Gel and PCR Clean-up Kit	Macherey-Nagel GmbH & Co. KG (DE)	Cat#REF740609.50
Phusion Hot Start II High-Fidelity DNA Polymerase	Thermo Fisher Scientific Inc. (US)	Cat#F549S
Qiagen RNeasy Plus Mini kit	Qiagen	Cat#74004
Qiagen DNeasy Blood & Tissue Kits	Qiagen	Cat#69504

**Experimental models: Cell lines**		

Human: HEK293T cells	David Sabatini’s lab	N/A
Mouse: mouse embryonic fibroblasts (MEF)	Stephan Storch (University Medical Center Hamburg-Eppendorf)	N/A
Human: fibroblast BJ line	ATCC, UK	Control: BJ CRL-2522
Human: fibroblast 1096-01 line	Dr. Timothy Yu (Boston Children’s Hospital, Boston MA)	Patient 1: 1096-01
Human: fibroblast BR3075 line	Dr. Benjamin Greenberg (UT Southwestern Medical Center, Dallas TX)	Patient 2: BR3075
Human: fibroblast BR2986 line	Dr. Benjamin Greenberg (UT Southwestern Medical Center, Dallas TX)	Patient 3: BR2986
African green monkey: Vero cells	Kindly provided by the Max-von-Pettenkofer-Institut, Munich	N/A

**Oligonucleotides**		

Integrin α2-subdomain (human), NM_002203.4, se: TTAGCGCTCAGTCAAGGCAT	This paper	Invitrogen
Integrin α2-subdomain (human), NM_002203.4, as: TTGCTTCTGGGAGACCAACA	This paper	Invitrogen
Integrin β1-subdomain (human), NM_002211.4, se: GCCGCGCGGAAAAGATGA	This paper	Invitrogen
Integrin β1-subdomain (human), NM_002211.4, as: TTGAATTTGTGCACCACCCAC	This paper	Invitrogen
Discoidin Domain Receptor Tyrosin Kinase 2 (human) NM_001014796.3, se: TGCACCCGTTGATATGCCTC	This paper	Invitrogen
Discoidin Domain Receptor Tyrosin Kinase 2 (human) NM_001014796.3, as: GAGTCCAGCCAAAGGTCTCC	This paper	Invitrogen
Tenascin C (human), XM_017014678.2, se: TCTCGCCCATCGGAAAGAAAA	This paper	Invitrogen
Tenascin C (human), XM_017014678.2, as: GGCTCTAGGGCTCTAGGGTAT	This paper	Invitrogen
Syndecan 3 (human), NM_014654.4, se: GAGGTGCTCGTAGCTGTGATT	This paper	Invitrogen
Syndecan 3 (human), NM_014654.4, as: AGCAGTGTGACCAAGAAGGC	This paper	Invitrogen
GAPDH (human), se: CCCCGGTTTCTATAAATTGAGC	This paper	Invitrogen
GAPDH (human), as: CTTCCCCATGGTGTCTGAG	This paper	Invitrogen
CLN7/MFSD8 (mouse), se: CCCGGAAGCAGAGAATGGAG	This paper	Invitrogen
CLN7/MFSD8 (mouse), as: GGCCAGATGGACATTATCAC	This paper	Invitrogen
CLN7/MFSD8 (human), se: CACCTGGAAGCAGAGAATGG	This paper	Invitrogen
CLN7/MFSD8 (human), as: ACCCTACACTGCTGAGAAAC	This paper	Invitrogen
single guide RNA (sgRNA) that contains a targeting sequence for CLN7 gene:		
sg CLN7_4 (S): caccgCTGGGCCAGATGAATCACCG	This paper	N/A
sg CLN7_4 (AS): aaacCGGTGATTCATCTGGCCCAGc	This paper	N/A
sg CLN7_9 (S): caccgTAGGCGACACACCTGGAAGC	This paper	N/A
sg CLN7_9 (AS): aaacGCTTCCAGGTGTGTCGCCTAc	This paper	N/A
CLN7-PAM-2-F: CCTGGTGCCTCTACACACCGGTG ATTCATCTGG	This paper	Metabion
CLN7-PAM-2-R: CCAGATGAATCACCGGTGTGTAGA GGCACCAGG	This paper	Metabion
SARS-CoV-2 E gene E_Sarbeco_F1: 5′-ACAGGTACGTT AATAGTTAATAGCGT-3′	Metabion international, Planegg (DE), N/A	Corman et al., (2020)
SARS-CoV-2 E gene E_Sarbeco_R2: 5′-ATATTGCAGCA GTACGCACACA-3′	Metabion international, Planegg (DE), N/A	Corman et al., (2020)
SARS-CoV-2 E gene E_Sarbeco_P1: FAM-ACACTAGCC ATCCTTACTGCGCTTCG-BBQ	Metabion international, Planegg (DE), N/A	Corman et al., (2020)
CLN7-EX2, se: 5′GAAACGGAGGGAGGAAGACA-3′	This paper	Invitrogen
CLN7-Ex2-In, as: 5′-CACCAGACTCAGGAGCCC-3′	This paper	Invitrogen
CLN7-EX11, se: 5′-TTTAACAGGATTGGCGAGCG-3′	This paper	Invitrogen
CLN7-EX11, as: 5′-ACCACAATGCCACTCCTACA-3′	This paper	Invitrogen

**Recombinant DNA**		

pIRES2-AcGFP1 vector	Clontech Europe	Cat#632435
pLentiCRISPRv1	David Sabatini’s lab	N/A
ΔVPR lentiviral packaging plasmid	Addgene	8455
VSV-G envelope plasmid	Addgene	8454

**Software and algorithms**		

ZEN Lite 2011 x64	Carl Zeiss AG, DE	https://www.zeiss.com/microscopy/int/products/microscope-software/zen-lite.html
ImageJ 2.0	Open source	https://imagej.nih.gov/ij/download.html
FlowJo Software Version 10	FlowJo	https://www.flowjo.com/
BD Accuri C6 Software	BD Biosciences	https://www.bdbiosciences.com/en-us/products/software
SnapGene	GSL Biotech	http://snapgene.com

**Other**		

Leibovitz’s L-15-Gibco® medium	Thermo Fisher Scientific Inc. (US)	Cat#11570396
PBS	Capricorn Scientific GmbH (DE)	Cat#PBS-1A
Dulbecco's Phosphate Buffered Salin (DPBS)	Thermo Fisher Scientific Inc. (US)	Cat#J67802.K2
Minimum Essential Medium (MEM) with Earle salts and L-glutamine	Capricorn Scientific GmbH (DE)	Cat#MEM-A (500 mL)
Gibco® Advanced DMEM/F-12 (Dulbecco’s Modified Eagle Medium/Ham’s F-12)	Thermo Fisher Scientific Inc. (US)	Cat#12634028
Opti-MEM® I Medium	Thermo Fisher Scientific Inc. (US)	Cat#31985070
Dulbecco's Modified Eagle’s Medium	10% fetal calf serum (FCS), 0.3 mg/mL Glutamin, 200 U/ml Penicillin, 90 U/ml Streptomycin	Gibco, Darmstadt (DE), Cat# 41,966

### Resource availability

#### Lead contact

Further information about the protocols and requests for resources and reagents should be directed to and will be fulfilled by the lead contact, Markus Reichold (markus.reichold@ur.de).

#### Materials availability

CLN7 deficient HEK293T cells are available upon request from Nouf Nasser M Laqtom (nlaqtom@stanford.edu). SARS-CoV-2 virus isolate CA is available upon request from Barbara Schmidt (Barbara.Schmidt@klinik.uni-regensburg.de). All other materials and reagents described in this papers are available upon request from the lead contact.

### Experimental model and subject details

#### CLN7-deficient HEK293T cell line

Human CLN7 was depleted using the pLentiCRISPRv1 system. The following sense (S) and antisense (AS) oligo-nucleotides were cloned into pLentiCRISPRv1:

sgCLN7_4 (S): caccgCTGGGCCAGATGAATCACCG

sgCLN7_4 (AS): aaacCGGTGATTCATCTGGCCCAGc

sgCLN7_9 (S): caccgTAGGCGACACACCTGGAAGC

sgCLN7_9 (AS): aaacGCTTCCAGGTGTGTCGCCTAc.

Lentiviruses were produced by transfecting HEK293T cells with one of the lentiviral plasmids, pLentiCRISPRv1, in combination with the packaging plasmids, VSV-G envelope and the ΔVPR using X-TremeGene 9 Transfection Reagent. The culture medium was changed to DMEM supplemented with 30% inactivated fetal calf serum 16 h post transfection. The virus-containing supernatant was collected 36-48 h post transfection and then frozen at −80°C.

To generate knockout cells, HEK293T cells were seeded at a density of 2 x10^6^ cells/mL in DMEM containing 8 μg/mL polybrene and then transduced with lentivirus by centrifugation at 1150 xg for 45 min at 37°C. Transduced cells were selected using DMEM containing puromycin for 72 h. Cells were then single-cell sorted into 96-well plates containing 200 μL of DMEM supplemented with 30% inactivated fetal calf serum. Cell clones with the desired knockouts were identified by Illumina amplicon deep sequencing at Massachusetts General Hospital (Boston, MA).

#### Genotyping of HEK293T CLN7 knockout cells

To demonstrate that our HEK293T knockout cells indeed no longer express CLN7, we first determined CLN7 mRNA expression using qPCR. In HEK293T cells, line 99-4 (Figure S1A, middle bar) CLN7 mRNA could no longer be detected. In line 94-5 (Figure S1A, right bar), however, we were still able to detect approximately 30% CLN7 mRNA compared to wild-type cells (Figure S1A, left bar).

In further experiments, we isolated genomic DNA from both HEK293T knockout cell lines and examined the effects of the CRISPR/Cas9 system on the CLN7 gene by sequencing (Figure S1B). For line 94-5, we observed a deletion of 4 bases in exon 11, causing a frameshift and thus a premature stop codon at amino acid position 423. The resulting mRNA is most likely recognized as defective and efficiently degraded, so that no CLN7 mRNA can be detected in this cell line. In line 99-4, a deletion of 6 bases occurs directly at the boundary between exon 2 and the following intron. Most likely, this causes a stop codon from the intron to enter exon 2, resulting in premature termination of the CLN7 protein. Alternatively, splicing could be affected. The resulting mRNA does not appear to be degraded quite as efficiently as in cell line 94-5, allowing some CLN7 mRNA to still be detected in qPCR using the primers described in the key resources table. Since there are no well-functioning antibodies against CLN7 available for purchase, knockout cannot be shown at the protein level.

### Method details

#### Genotyping of HEK293T CLN7 knockout cells

Genomic DNA was extracted from HEK293T wild-type and CLN7-deficient cells (lines 94-5, 99-4) using a Qiagen DNeasy Blood & Tissue Kit according to the manufacturer’s protocol. The DNA quality was assessed by optical absorbance and gel electrophoresis. Exon 2 and the exon/intron junction of exon 11 of the CLN7 gene were amplified by PCR and sequenced using the Sanger method with the following primers: CLN7-EX2 (se), CLN7-Ex2-In (as), CLN7-EX11 (se), CLN7-EX11 (as) (see key resources table). Sequencing data were analyzed using SnapGene software (GSL Biotech). The results are presented in Figure S1.

#### Infection of HEK293T cells with SARS-CoV-2

HEK293T wild-type and CLN7 knockout cells were plated in flat bottom 96-well plates at 15.000 cells/well. They were infected using the indicated doses of a SARS-CoV-2 wild-type strain (GenBank accession no. MZ675816), as described recently (Peterhoff et al., 2021). Viral stocks were grown and titrated in Vero cells, using the method of Reed and Munch (1938) to determine the 50% tissue culture infective dose (TCID_50_). To get rid of the input virus, supernatants were removed completely and replaced by fresh media 12-24 h post infection. Cell culture supernatants were harvested 48 h post infection and viral loads were analyzed using a quantitative SARS-CoV-2 reverse transcriptase PCR according to a published protocol (Corman et al., 2020) on the StepOnePlus Real-Time PCR System (ThermoFisherScientific, Schwerte, DE). In further experiments, we specifically analyzed the entry of SARS-CoV-2 via measuring SARS-CoV-2 RNA in cell pellets trypsinized at 12 h p.i. and normalizing viral loads for the internal housekeeping β-glucuronidase (GUS).

To control for unspecific binding of non-infectious SARS-CoV-2 particles to cells, cells were fixed with 4% paraformaldehyde for 1 h, washed five times with DBPS, and then infected with SARS-CoV-2 at the same MOI as unfixed cells. Subsequently, the viral loads in cell culture supernatants and cell pellets were compared between fixed and unfixed cells.

#### GM1 quantification and staining on HEK293T cells

Quantification of ganglioside GM1 in CLN7-deficient cells (wild-type and line 99-4) was performed by flow cytometry. Alexa Fluor(AF) 647nm-coupled subunit B of cholera toxin (CTxB) was used as a dye. CTxB binds specifically to GM1 enriched in lipid rafts and shows no cell toxic properties (Heyningen, 1974). For the experiments, 600,000 cells were seeded in 6-well plates. Two days later cells were incubated with 1 mL staining solution (10 μg/mL) for 30 min at 37°C. Control groups (unstained cells) were treated with 1 mL of Leibovitz’s medium without CTxB. Subsequently, all cells were washed with pre-warmed Leibovitz’s medium and detached by adding 1 mL accutase solution (1:5 in DPBS, 5 min at room temperature). After centrifugation (2,000 rpm, 3 min), cell pellets were taken up in 800 μL of cold Leibovitz’s medium and kept in the dark and on ice until measurement. Measurements were performed on a BD Accuri C6 from BD with an excitation wavelength of 640 nm and the FL4 filter (675/25 nm) for the emitted light. The FSC-H threshold was set to 80,000. Three dishes from each experimental group (HEK293T wt and CLN7 ko 99-4) were examined during the day, and each of these samples was measured in triplicate. Measurements were made on five consecutive days. The background signal of unstained cells was not subtracted because it was very faint compared to the signal of stained cells. At least 10,000 events were recorded. The median of the fluorescence signal was evaluated.

For microscopy, CTxB was diluted with Leibovitz’s L-15-Gibco medium to a working concentration of 10 μg/mL. In addition, phalloidin-AF647 (1:400) was added to the staining solution, which labels F-actin to visualize the cells. Cells cultured on glass plates were incubated with 1 mL staining solution for 30 min in an incubator. This was followed by a washing step with 1 mL each of pre-warmed Leibovitz’s medium before live cell imaging was performed.

#### Generation of vector with PAM mutation for HEK293T 99-4 knockout cell line

Full-length CLN7 (NM_001371596.2; 1557 bp in length encoding 518 amino acids) was cloned into the pIRES-GFP vector using XhoI/BamHI restriction sites. Mutation in position T411 (ACC>ACA) of CLN7 was introduced in order to disrupt PAM sequence for the HEK239T 99-4 knockout cells, using the following primers: CLN7-PAM-2-F; CCTGGTGCCTCTACACACCGGTGATTCATCTGG, and CLN7-PAM-2-R; CCAGATGAATCACCGGTGTGTAGAGGCACCAGG. The final CLN7 construct was fully sequenced to ensure the targeted mutagenesis had occurred correctly and to exclude the presence of undesired sequence alterations.

#### GM1 measurements on patient’s fibroblasts

The fibroblasts used in this study were obtained from Dr. Timothy Yu (Boston Children’s Hospital, Boston MA) and Dr. Benjamin Greenberg (UT Southwestern Medical Center, Dallas TX) labs, and were maintained and passaged in DMEM media supplemented with 10% Fetal Bovine Serum. Fibroblasts only under passage 20 were used for experiments. Fibroblasts were plated at 1 × 10^5^ cells/well in 24-well plates. Two days after seeding, cells were fixed for 15 min in 4% paraformaldehyde in PBS (pH 7.4) at room temperature, followed by three PBS washes. GM1 gangliosides in the fixed cells were labeled with 1 μg/mL Alexa Fluor 488-conjugated Cholera Toxin Subunit B in PBS for 1 h at room temperature. Nuclei were stained with 4′,6-diamidino-2-phenylindole (DAPI) contained in mounting media.

Images were acquired on the Leica DMI4000B confocal microscope, and 10X and 40X objectives. Signal intensity measurement of cells containing CTxB-AF488 staining was performed using three to five arbitrarily selected fields per coverglass on three coverslips per line and on two different cell passages. The signal intensity of the CTxB-AF488 staining was normalized to the DAPI signal intensity. Each dot on the graph represents the normalized CTxB-AF488-fold change to control line (Control) per field.

#### Quantification of macropinocytosis activity in HEK293T cells

Quantification of macropinocytosis activity was performed using the high molecular weight sugar dextran (10 kDa) coupled to Alexa Fluor 546 nm. Dextran is taken up into cells mainly via macropinocytosis and has no cell toxic effects (Li et al., 2015). According to manufacturer’s protocol, dextran was first dissolved in PBS (5 mg/mL) and then diluted with cell culture medium to a final concentration of 0.5 mg/mL. Cells were seeded in 6-well plates and incubated with 1 mL staining solution for 60 min. As a control, 3 wells were incubated with just cell culture medium. After removing the excess staining solution, the cells were detached with 1 mL trypsin-EDTA (5 min, 37°C), transferred to 2 mL cell culture medium, and centrifuged at 1,000 rpm for 3 min. The cell pellet was resuspended in 400 μL FACS buffer (2% FCS in DPBS), and samples were stored on ice in the dark until measurement. Flow cytometry was performed on a BD FACSCelesta flow cytometer from BD. Before measurements cell suspensions were filtered through a 35 μm cell strainer to avoid cell clumps. At least 10,000 events were recorded from each sample. BD’s FACSDiva software was used for measurements, and FlowJo was used for analysis. Three dishes from each experimental group (HEK293T wt and CLN7 ko 99-4) were examined per day, and each of these samples was measured in triplicate. Measurements were made on three consecutive days. The median of the fluorescence signal was evaluated.

For microscopy, 500,000 - 800,000 cells were seeded on glass cover slips. The next day, cells were incubated with 1 mL staining solution for 1 h at 37°C. Then, they were washed with pre-warmed DPBS to remove excess staining solution. Live cell microscopy was performed using an inverted microscope from Zeiss (Zeiss Observer.Z1). Images were taken with a 63x objective. Zeiss filter set 43 was used (545 BP/25, FT 570, 605 BP/70). For live cell microscopy, cells were covered with pre-warmed colorless Leibovitz’s L-15-Gibco medium and kept at a temperature of 37°C.

#### Measurement of cell migration in HEK293T cells (neurosphere assay)

The neurosphere assay is a test for the undirected migratory ability of a cell. In this assay, cells are cultured in wells coated with agarose. Unable to establish adhesion with the substrate, they assemble into their most thermodynamically and mechanically stable form, a sphere (Li et al., 2015). If these spheres are now applied to a surface to which they can adhere, they will attempt to grow out centrifugally and migrate. By coating them with specific molecules, the migratory ability of the cells on different surfaces can be studied. For this purpose, the area of the outgrowing cell spheres must be measured directly after seeding and after defined periods. The migration ability of the cells represents a function of the increase in area over time (Marshall et al., 2007).

96-well plates were coated with 100 μL of 1% agarose. Then, 5000 cells each were seeded in a volume of 200 μL/well and incubated for 48 h at 37°C and 5% CO_2_. In parallel, the well plates were coated with different proteins of the extracellular matrix: Laminin was coated at 10 μg/cm^2^ for 2-3 h at 37°C. Fibronectin was applied at a concentration of 2–5 μg/cm^2^, immediately removed for the well and air-dried for at least 60 min. Collagen I was used at a concentration of 6–10 μg/cm^2^ and incubated for several hours at room temperature. Subsequently, the liquid was removed and the well plate was dried overnight. A rinse with culture medium was performed before seeding the cells. Collagen IV was used at a concentration of 10 mg/cm^2^ for coating. Wells were incubated overnight with the coating medium. The following day, the solution was removed and the wells were dried overnight.

The next step was to transfer the spheres into the coated 96-well plates, each containing 100 μL of cell culture medium. For the 0 h value, pictures of the spheres were taken immediately after the transfer. Additional pictures were taken after 3, 19, 25 and 43 h. To assess the migration ability, a determination of the area of outgrowing cells was performed using ZEN Lite 2011 x64 software (Carl Zeiss AG, DE). For the analysis, the 0-h value of the respective sphere was subtracted. Ten neurospheres per coating were examined per day from each experimental group (HEK293T wt, CLN7 ko 94-5 and 99-4) and the values were averaged. The experiments on HEK293T wt and the knockout cell line 99-4 were performed on five consecutive days, whereas the experiments on knockout cell line 94-5 were performed on three days.

#### Measurement of cell adhesion in HEK293T cells

Cell adhesion was quantified using a flow chamber assay (Hafezi-Moghadam et al., 2004). For this purpose, uncoated μ-Slides VI 0.4 from Ibidi-Systems were coated with Fibronectin according to the manufacturer’s protocol. The chambers were filled bubble-free with cell culture medium (Modified Eagle’s Medium with Earle salts and L-glutamine, 10% FCS, 1% penicillin/streptomycin) and pre-warmed in an incubator. Cells were then detached with trypsin and diluted to a concentration of 2.5x10^6^ cells/ml. Approximately 200 μL of this suspension was pipetted into the coated chambers. Cells were then cultured in an incubator (37.0°C, 5.0% CO_2_) for approximately 3 h until adhesion took place. An attempt was made to measure all cells at the same degree of confluency.

To quantify the cells before the experiment, they were stained for half an hour with the nuclear dye Hoechst 33,342 (0.1 μg/mL in Leibovitz’s medium). A first image of a 2500 × 2500 μm area of the chamber bottom was taken with a 10x objective. The filter set 49 from Zeiss was used (Excitation G 365, Emission 445 BP/50).

Subsequently, the cells were subjected to a mechanical force using a fluid stream. Ringer’s solution was used as the perfusion fluid (145 mM NaCl, 3.6 mM KCl, 5 mM HEPES, 5 mM glucose, 1 mM MgCl_2_, 1.3 mM CaCl_2_, pH7.4). After a perfusion time of 5 min at a flow rate of 50 mL/min, another image was obtained from exactly the same area. The nuclear area was quantified using ImageJ 2.0 software, which is proportional to the number of cells. Dividing the nuclear area after by the nuclear area before application of the flow yields a quotient (adhesion quotient Q) that is proportional to the adhesion ability of the cells. A large Q close to 1,0 means that adhesion of the cells is high. Four chambers from each experimental group (HEK293T wt, CLN7 ko 94-5 and 99-4) were examined per day. The experiments were performed on three consecutive days.

#### qPCR

For isolation of mRNA from cells, the RNA isolation kit NucleoSpin was used according to the manufacturer’s protocol. The cDNA synthesis from the RNA was performed using the Reverse Transcription System from Promega Corporation (US). The protocol followed was also in accordance with the manufacturer’s recommendations. The reaction mixture for the qPCR consisted of 10 μL Takyon No Rox SYBR MasterMix dTTP Blue, 0.5 μL each of a sense (se)/antisense (as) primer (see key resources table) for the respective gene, 3.5 μL water and 1 μL of the template. This master mix included a hotstart DNA polymerase, dNTPs, MgCl2, buffer, and the fluorescent dye SYBRgreenI.

The reaction solutions were pipetted into 96-well plates. The instrument used was a LightCycler 480 Instrument II (96-well) from F. Hoffmann-La Roche AG (CH). Forty cycles were performed. One cycle consisted of 3 min activation of the polymerase at 95°C, 15 s denaturation phase at 95°C, 20 s annealing at 57°C and an elongation phase of 20 s at 72°C.

#### Quantification of ACE2 surface expression in HEK293T cells

To examine ACE2 surface expression in our CLN7-deficient cell model, an APC-coupled IgG polyclonal antibody was used. An isotype control was done to determine background signal by nonspecific antibody binding.

For staining, cells were detached with accutase. After centrifugation (2,000 rpm, 3 min), cells were resuspended in 1 mL of cold FACS buffer (2% FCS in DPBS) and stored on ice. 800,000 cells from each experimental group were harvested and transferred to 50 μL of FACS buffer each. For the staining solution, 10 μL of ACE2 or isotype control antibody was added to 50 μL of FACS buffer. All samples were then incubated at 4°C protected from light for 30 min. Cells were then washed with 1 mL FACS buffer, resuspended in 300 μL FACS buffer, and kept on 4°C until measurement.

Measurements were performed on a BD Accuri C6 from BD with an excitation wavelength of 640 nm and the FL4 filter (675/25 nm) for the emitted light. At least 10,000 events were recorded. The median of the fluorescence signal was evaluated. The signal of the isotype control antibody was subtracted from the fluorescence signal of the ACE2 antibody. Three dishes from each experimental group (HEK293T wt, CLN7 ko 94-5, and 99-4) were examined per day. The cells of each dish were measured once and the mean value for one day was calculated from them. The experiments were performed for nine consecutive days. The median of the fluorescence signal was evaluated.

#### Electron microscopy

For electron microscopy studies, HEK293T wild-type and CLN7 knockout cells were infected with SARS-CoV-2 for 48 h according to the protocol described above. Cells were then fixed with 3% paraformaldehyde solution (in PBS) for 24 h and then transferred to a 2% glutaraldehyde solution (in 0.1 M sodium cacodylate, pH7.4) for approximately 12 h. Dehydration of the cell pellets was performed according to the following protocol: First, cell pellets were embedded in 1.5% agarose in 0.1 M sodium cacodylate buffer. This was followed by a washing step with 0.1 M sodium cacodylate buffer (3 × 20 min), 1% osmium tetroxide in 0.1 M sodium cacodylate buffer (2 h), 0.1 M sodium cacodylate buffer (3 × 20 min), 50% ethanol (15 min), 70% ethanol (15 min), 90% ethanol (15 min), 96% ethanol (15 min), 100% ethanol (20 min), acetone (3 × 15 min). The pelletized cells were then embedded in Epon at 60°C for 48 h according to standard protocol. To increase contrast, samples were treated with 1% uranyl acetate for 30 min and lead citrate for 1 min. Examination of the sections was performed on an EM902 (Zeiss, Oberkochen, Germany) transmission electron microscope. Digital images were obtained using a 2k CCD camera (Troendle, Moorenweis, Germany).

### Quantification and statistical analysis

Data are shown as mean values ±SEM One-way ANOVA with Tukey’s post-hoc test was used to calculate significance between different groups. A p value ≤ 0.05 was accepted to indicate statistical significance, which was identified by an asterisk (∗). Statistical calculations were performed with GraphPad Prism, Version 9.0 or with Origin 2020.

## Data Availability

• All data reported in this paper will be shared by the lead contact upon request. • This paper does not report original code. • Any additional information required to reanalyze the data reported in this paper is available from the lead contact upon request.
