# Supplementary material for: CLN7/MFSD8 may be an important factor for SARS-CoV-2 cell entry
Source: iScience. 2022 Sep 6;25(10):105082. doi: 10.1016/j.isci.2022.105082 (PMC9444308; doi:10.1016/j.isci.2022.105082)
Supplement: Document S1. Figures S1 [file mmc1.pdf]

iScience, Volume *25*

Supplemental information

CLN7/MFSD8 may be an important factor for SARS-CoV-2 cell entry

Elena-Sofia Heinl, Sebastian Lorenz, Barbara Schmidt, Nouf Nasser M Laqtom, Joseph R. Mazzulli, Laetitia Francelle, Timothy W. Yu, Benjamin Greenberg, Stephan Storch, Ines Tegtmeier, Helga Othmen, Katja Maurer, Malin Steinfurth, Ralph Witzgall, Vladimir Milenkovic, Christian H. Wetzel, and Markus Reichold

Figure S1: Genotyping of HEK293T CLN7 knockout cells, Related to STAR Methods

a)

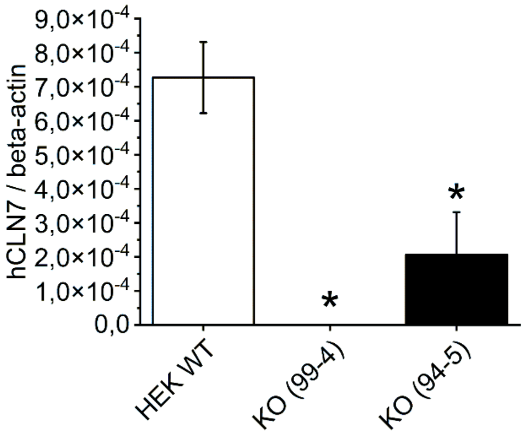

b)

Line 94-5: Exon 11 (CLN7 genomic DNA NG_008657.1)

WT:

```
408                                     423
 L  Y  T  P  V  I  H  L  A  Q  F  L  T  S  A  V
CTCTACACCCCGGTGATTCATCTGGCCCAGTTCCTTACATCAGCTGTG
```

94-5: GGTG deleted and frameshift from P411

```
408                                     423
 L  Y  T  P  F  I  W  P  S  S  L  H  Q  L  C  *
CTCTACACCCCATTCATCTGGCCCAGTTCCTTACATCAGCTGTGTTAA
```

>> Frameshift leads to a premature stop codon in exon 11

Line 99-4: Exon 2 (CLN7 genomic DNA NG_008657.1)

WT:

```
...................exon......................V..intron..
 12                                   20
 L  L  G  D  T  P  G  S  R
CTCTTAGGCGACACACCTGGAAGCAGGTGA
```

99-4: AGCAGG deleted

```
...................exon........................
 12                             19
 L  L  G  D  T  P  G  *
CTCTTAGGCGACACACCTGGATGA
```

>> Deletion leads to a shift of a **stop codon** from intron to previous exon 2 or splicing is affected

a) Measurement of CLN7 mRNA expression in our CLN7-deficient HEK293T cell lines using quantitative PCR. In line 99-4 (middle bar) CLN7 mRNA could no longer be detected. In line 94-5 (right bar), approximately 30% CLN7 mRNA could still be detected compared to wild-type cells (left bar). b) Genomic DNA from both HEK293T knockout cell lines was isolated, amplified and sequenced. For line 94-5, we observed a deletion of 4 bases in exon 11, causing a frameshift and thus a premature stop codon at amino acid position 423. In line 99-4, a deletion of 6 bases occurs directly at the boundary between exon 2 and the following intron. Most likely, this causes a stop codon from the intron to enter exon 2, resulting in premature termination of the CLN7 protein. Alternatively, splicing could be affected.
